# Clinical Trial Endpoints and Their Clinical Meaningfulness in Early Stages of Alzheimer’s Disease

**DOI:** 10.14283/jpad.2022.41

**Published:** 2022

**Authors:** S. Cohen, J. Cummings, S. Knox, M. Potashman, J. Harrison

**Affiliations:** 1. Toronto Memory Program, Toronto, ON, Canada; 2. Chambers-Grundy Center for Transformative Neuroscience, Department of Brain Health, School of Integrated Health Sciences, University of Nevada, Las Vegas (UNLV), NV, USA; 3. Biogen International GmbH, Baar, Switzerland; 4. Biogen Inc., Cambridge, MA, USA; 5. Metis Cognition Ltd, Wiltshire, UK; 6. Alzheimer Center, AU Medical Center, Amsterdam, the Netherlands; 7. Institute of Psychiatry, Psychology & Neuroscience, King’s College London, UK

**Keywords:** Early Alzheimer’s disease, clinical meaningfulness, clinical trials, endpoints, assessment tools

## Abstract

As the focus of Alzheimer’s disease (AD) therapeutic development shifts to the early stages of the disease, the clinical endpoints used in drug trials, and how these might translate into clinical practice, are of increasing importance. The clinical meaningfulness of trial outcome measures is often unclear, with a lack of conclusive evidence as to how these measures correlate to changes in disease progression and treatment response. Clarifying this would benefit all, including patients, care partners, primary care providers, regulators, and payers, and would enhance our understanding of the relationship between clinical trial endpoints and assessments used in everyday practice. At present, there is a wide range of assessment tools used in clinical trials for AD and substantial variability in measures selected as endpoints across these trials. The aim of this review is to summarize the most commonly used assessment tools for early stages of AD, describe their use in clinical trials and clinical practice, and discuss what might constitute clinically meaningful change in these measures in relation to disease progression and treatment response.

## Introduction

Alzheimer’s disease (AD) is a neurodegenerative disorder accounting for ~60–80% of late-onset dementia cases worldwide (1). It represents an enormous disease burden for patients, care partners, healthcare institutions and society at large (1). The main clinical features of AD are progressive impairments of cognition and function and changes in behavior (2, 3). It has been postulated that the optimal opportunity to meaningfully impact the course of AD, reduce disease burden and preserve function, may be prior to substantial clinical symptom appearance when the underlying pathology is limited (4).

The current treatment development landscape in AD includes therapeutic candidates that target the underlying pathophysiology of AD in the hope of significantly altering the course of the disease (5). Many of these are being investigated as potential treatments for the early stages of AD; 12/29 Phase 3 trials (41.4%) and 37/73 Phase 2 trials (50.7%) involve patients with mild cognitive impairment (MCI) due to AD or prodromal-to-mild AD dementia (5). In these trials, certain clinical measures such as Clinical Dementia Rating̱–Sum of Boxes (CDR-SB), Alzheimer’s Disease Assessment Scale–Cognitive Subscale (ADAS-Cog), and Alzheimer’s Disease Cooperative Study–Activities of Daily Living Scale–Mild Cognitive Impairment (ADCS-ADL) are often used as trial outcomes (5), but a variety of factors (including practical limitations such as long administration times and limited sensitivity to change in early stages of the disease) limit their use outside clinical trials in the diagnosis and disease monitoring of patients with AD. As new therapeutic options emerge for the treatment of AD, such as aducanumab, which was recently granted accelerated approval for the treatment of AD by the US Food and Drug Administration (FDA), it is important that the clinical meaningfulness of the endpoints used to evaluate these therapeutics in clinical trials is understood. Firstly, there is a need to evaluate the totality of evidence from both clinical and biomarker effects to make an accurate estimate of effect on disease progression (6). It is critical to have a scientifically justified approach to assess the significance and persistence of the potential impact of a therapeutic along the disease course (6). Secondly, there needs to be a pragmatic focus on endpoint selection in clinical trials, considering the overlap of cognitive, functional, and behavioral changes, and the differences in sensitivity of these endpoints in detecting changes along the disease course, especially in the early disease stages (7–9). Finally, there is a need for translation of clinical trial outcome data to inform decision making in clinical practice, from the perspectives of healthcare practitioners, payers, patients, and care partners.

Here we review commonly used clinical trial endpoints and their applicability in clinical practice. We also examine clinically meaningful changes in the outcome measures in early stages of AD, focusing on those used in MCI due to AD and mild AD dementia.

## Key clinical features assessed in AD clinical trials

The three main clinical features commonly assessed in AD clinical trials are cognition, function, and behavior (3). These can be assessed separately or through composite or global assessments (2, 3). Clinical presentation of AD decline can vary among individuals (Table 1) (10).

**Figure 1.**
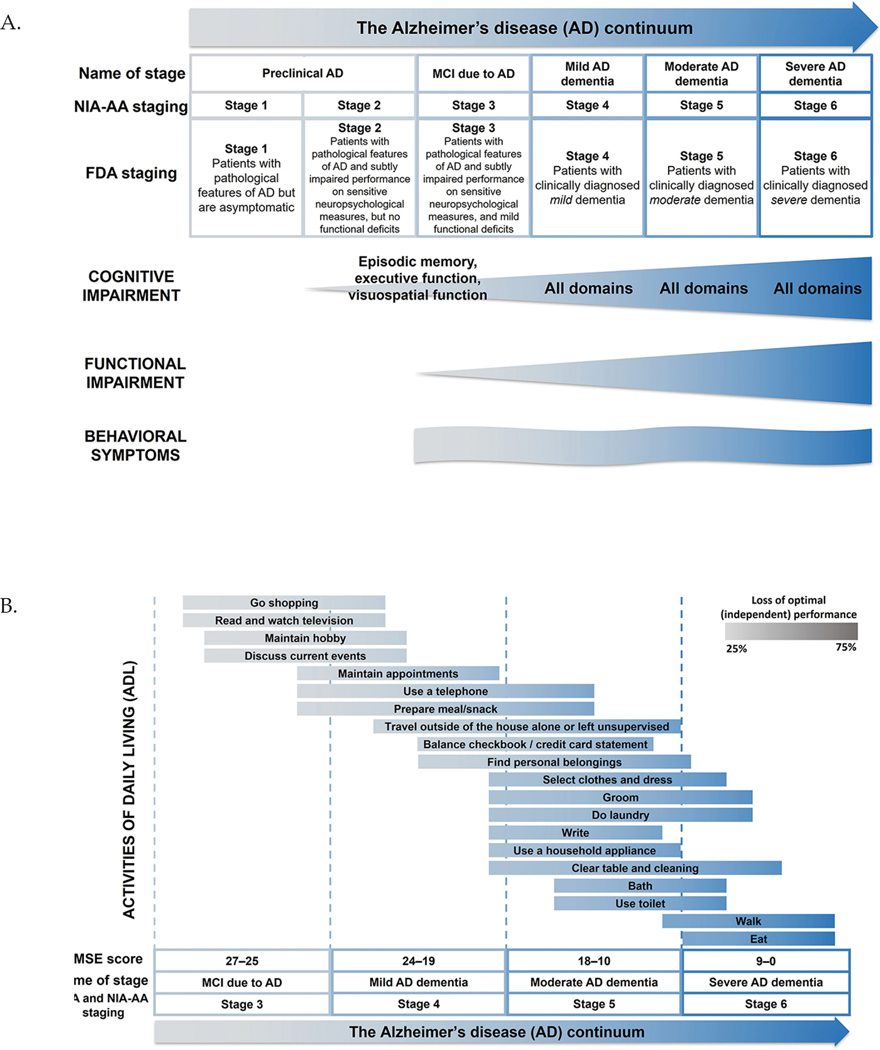
Cognitive, functional, and behavioral impairment across the AD continuum A. There is progressive cognitive and functional decline, initially observed in Stages 2 and 3 of AD, as classified by the FDA in 2018 (U.S. Food and Drug Administration 2018). Behavioral impairment differs across the continuum, with variable appearance and resolution of behavioral symptoms. B. Different ADL are impaired differentially across the AD continuum, with each ADL being initially affected during different stages of AD. Adapted from Galasko 1998 with permission (Galasko 1998). Abbreviations: AD = Alzheimer’s disease; ADL = activities of daily living; FDA = Food and Drug Administration; MCI = mild cognitive impairment; MMSE, Mini-Mental State Examination; NIA-AA= National Institute of Aging–Alzheimer’s Association

In the assessment of cognition, multiple domains are evaluated, including attention, memory, executive function, language, visuospatial processing, and praxis (Table 1), but there is limited consensus on the discreteness of these functions (11). Cognitive decline impacts all cognitive domains to a variable extent and is associated with increasing functional impairment over time (Table 1; Figure 1A, 1B) (11–14).

The assessment of function describes a patients’ ability to perform activities normally associated with everyday life. Function can be assessed through the performance of basic activities of daily living (BADL), which involve activities required for personal self-maintenance (Figure 1B) (8), and instrumental activities of daily living (IADL), which involve higher-level activities, such as making a meal or cleaning a room. IADL place greater demand on cognitive resources and therefore represent a more sensitive outcome to assess early functional loss than BADL (Figure 1B) (8). Notably, decline in IADL can be masked by compensatory mechanisms in very early stages of AD, presenting a challenge for accurate and complete clinical assessment (15).

Behavior in AD is assessed through presence and severity of neuropsychiatric symptoms (NPS) (Figure 1; Table 1) (7, 16, 17). NPS worsen with cognitive impairment (7) and the presence of NPS may predict further cognitive decline, decreased functioning, increased care partner burden, increased healthcare utilization and costs, earlier need for institutionalization, and increased risk of death (16–19). However, patient-to-patient variability exists in the occurrence and severity of NPS (17), so cognition can serve only as a rough proxy for progressive decline in function and behavior in individuals with AD. Evaluation of all three clinical features is key for comprehensive clinical assessment of disease stage, progression, and potential treatment response.

## Key clinical outcomes of interest across the AD continuum

The updated National Institute of Aging–Alzheimer’s Association (NIA-AA) research framework proposed to classify individuals along the continuum of AD, based on the presence of biomarkers of AD neuropathology (4). Cognition, function, and behavior are affected differentially across the AD continuum (Figure 1A, 1B) (20). According to the latest guidance from the FDA, during Stage 1, there is no objective evidence of cognitive decline or functional impairment (20), although some individuals report subjective cognitive decline (20). During Stage 2, there is objective evidence of impairment observed in select cognitive domains, with preservation of IADL (13). Stages 3 and 4 represent MCI due to AD and mild AD dementia, respectively. During Stage 3, one or more of the cognitive domains are affected and there are difficulties with word-finding (a component of the language domain) (11, 13). During Stage 4, multiple cognitive domains are affected and there is increased cognitive and functional decline compared with Stage 3 (4). Notably, cognition and function are often correlated in Stage 4, as cognitive decline generally precedes functional decline (12, 14). However, this is not always the case, as many environmental variables, compensatory strategies, and assistive devices may ameliorate functional decline, without any accompanying improvement in cognition. While executive function may be the most relevant cognitive domain in determining function (21, 22), reliance on specific cognitive domains will vary depending on the demands of the IADL in question.

The focus of drug development is shifting to therapeutic interventions targeting early stages of AD. During MCI and mild AD dementia, patients tend to exhibit slow and variable progression, which can complicate disease assessment (9, 23). Patients may remain in Stage 3 for ~3–5 years and in Stage 4 for ~3 years, with the individual rate of decline influenced by age, sex, apolipoprotein E (APOE) genotype, and potentially other factors yet to be identified (24). The variable length of time during which an individual may remain in the early stages of disease poses challenges for detecting treatment response.

As the disease progresses to Stages 5 and 6, where moderate and severe AD dementia occur, all cognitive domains are increasingly impaired, with greater functional declines in IADL, and progressive deterioration in BADL (Figure 1B) (4, 8). While cognition and function decline continuously, behavioral symptoms vary throughout the disease, with apathy being among the most common symptoms in early stages of AD, followed by aggression and agitation in later stages (Figure 1A) (17).

## Clinical trial endpoints for early stages of AD

**Figure 2.**
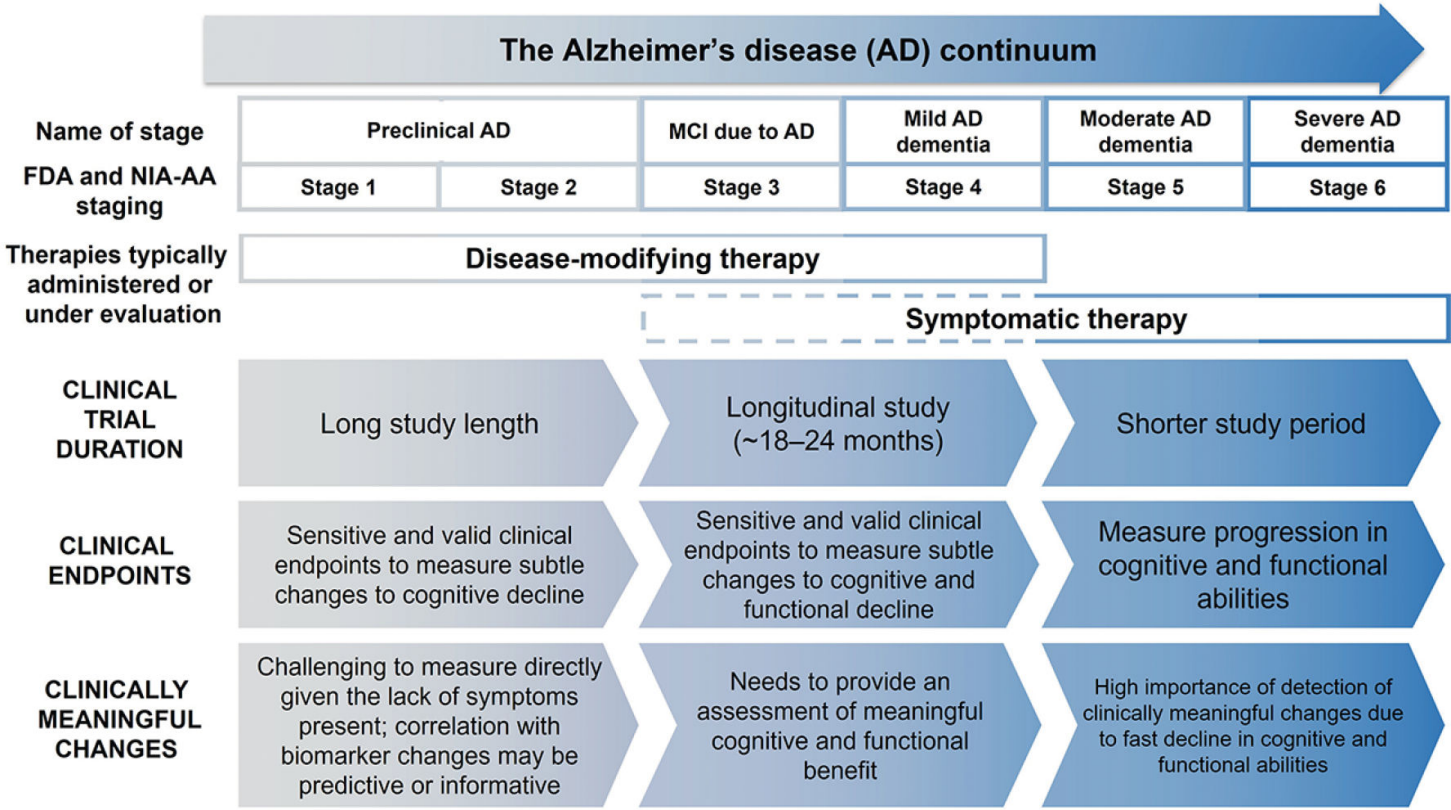
Selection of clinical endpoints depends on the stage of Alzheimer’s disease Clinical trials for AD vary in length based on the population of patients they involve, as classified here by the FDA staging (U.S. Food and Drug Administration 2018). Selection of clinical endpoints for clinical trials differs dependent upon the clinical stage of AD, with measurement of clinically meaningful changes also having varying challenges across the AD continuum. Abbreviations: AD = Alzheimer’s disease; FDA = Food and Drug Administration; MCI = mild cognitive impairment; NIA-AA = National Institute of Aging–Alzheimer’s Association

Historically, clinical trial endpoints have been designed to measure symptom changes in patients with mild-to-moderate or severe AD dementia (25, 26). With the shift of focus toward developing AD therapeutics targeting MCI due to AD and mild AD dementia, it has become necessary to design and validate clinical trial endpoints sensitive to changes that occur during these stages (Figure 2) (5, 25, 26). Changes in MCI due to AD and mild AD dementia are challenging to capture due to high variability in baseline functioning and cognitive ability, and the floor and/or ceiling effects of many of the clinical assessment tools that evaluate AD, which may limit their utility across the continuum of disease (9). These changes may be difficult to notice, depending on patient’s lifestyle (9) and potential compensatory mechanisms (27), but can be meaningful to the individuals experiencing them. Measures used in clinical trials for MCI due to AD and mild AD dementia must be sensitive enough to differentiate continuing decline in the placebo group from slowed progression in response to active treatment (25).

A variety of tools assessing clinical features are used as primary or secondary endpoints in clinical trials for MCI due to AD and mild AD dementia. These include measures of cognition, function, and behavior, as well as global change, quality of life (QOL), and care partner burden (Table 2A) (2, 3, 5, 28–42). Both the European Medicines Agency (EMA) and FDA have provided guidance on endpoints for AD trials, dependent on disease stage (20, 43). When selecting appropriate assessment tools for clinical trials, several factors should be considered: baseline cognitive severity, test validity, test sensitivity and specificity, test completion time, test-retest reliability, inter-rater reliability, global multi-site setting application, and ability to show clinically meaningful changes (3, 28). These measures for patients with MCI due to AD and mild AD dementia have differing levels of psychometric performance data (Table 2A). Patients with early AD have subtle cognitive deficits and do not present with functional impairment; patients who are closer to the onset of dementia may have noticeable functional deficits that progress slowly, creating sensitivity issues with currently available scales. Furthermore, the extent to which an individual compensates for cognitive deficits and adjusts for daily activities is very variable. It is thus challenging to establish a clinically meaningful effect during a trial of reasonable duration. Both guidelines suggest an alternative time-to-event approach (e.g., time to the occurrence of a clinically meaningful event during disease progression) to evaluate beneficial effects in trials in early AD. While independent assessment of daily function and cognitive effects may be acceptable, the FDA guideline states that measurable cognitive benefit should not allow for an overall finding of efficacy in the absence of meaningful functional benefit, and vice versa. The EMA guideline states that measures of cognition and function may be included as secondary endpoints to contribute to the overall assessment of efficacy. Both guidelines agree that the use of multiple individual tests may increase the persuasiveness of an overall finding of efficacy. There is a need to construct more sensitive rating scales involving domains that have been shown to be impaired consistently in early AD.

Complementary to clinical measures, imaging and fluid biomarkers quantify measures of brain characteristics or peripherally available markers of pathological changes due to AD, even at early stages when clinical measures might have limited sensitivity. Robust evidence supports the utility of biomarkers to predict the likely trajectory of clinical progression (2), and as such, should be evaluated in conjunction with clinical features to provide a comprehensive estimate of ongoing disease progression (4).

### Ability of clinical endpoints to detect changes in clinical features in early stages of AD

#### Detection of changes in cognition in early AD

As cognition includes a broad spectrum of processes, capturing its full breadth with any one assessment tool is challenging. The EMA guidance on designing AD trials stipulates that specific components of currently available tools are sensitive to detection of disease progression in MCI and mild AD dementia (43). Episodic memory, executive function, and visuospatial function, which are known to be most affected during MCI due to AD (Figure 1A), using tools that focus on these functions may therefore be most appropriate for use in early stages of AD (9, 13, 31). Appropriate cognitive assessment tools that can measure longitudinal changes, especially subtle shifts as observed in MCI, are critical to understanding disease progression and determining treatment effects in longitudinal studies (44).

The Mini-Mental State Exam (MMSE) and the Montreal Cognitive Assessment (MoCA) are similar cognitive assessments tools; both are brief and examine orientation, immediate and delayed recall, language abilities, attention, and visuospatial ability (5). MoCA has more measures of executive function. Due to the floor and ceiling effects of the MMSE, low sensitivity in early stages of AD, and test items weighted heavily toward the memory domain, with minimal assessment of other domains (language, visuospatial function, and executive function), the MoCA may be more appropriate for use in early stages of AD (45). However, although commonly used in clinical practice, the MoCA is not widely used in clinical trials, whereas the MMSE is used in both (Table 2A) (5). The MMSE may be the preferred tool in longitudinal studies as the MoCA may be a more challenging test (that includes executive function, complex visuospatial processing, and higher-level language) for patients with late-stage AD, where cognitive function is more severely impaired (46).

Another tool used widely in clinical trials for mild-to-moderate AD dementia is the ADAS-Cog, which assesses the cognitive domains of memory, language, and praxis (45). The standard ADAS-Cog-11 suffers from ceiling effects at mild stages of AD (47, 48), whereas the modified ADAS-Cog-13, through the addition of a delayed word recall task and a number cancellation task, has an increased sensitivity in mild AD dementia compared with the ADAS-Cog-11. However, sensitivity to change in MCI due to AD remains limited (Table 2A) (48). Additional tools used in AD clinical trials or clinical practice include the Neuropsychological Test Battery (NTB) (32) and the Repeatable Battery for the Assessment of Neuropsychological Status (RBANS) (Table 2A) (28, 30). These tools, and a variety of others, have been translated and validated in multiple languages (28, 49, 50). However, for broad application, global interpretation of results and sensitive detection of changes in early AD, assessment tools should be validated in populations of diverse educational and cultural backgrounds (9, 28).

The cognitive scales discussed above are composite measures of multiple cognitive domains comprising a global cognitive measure. However, as new compounds may have differential effects across cognitive domains, this approach may represent an oversimplification (2). A detailed discussion is beyond the remit of this paper, but interested readers are directed to a recent review on this topic (2).

#### Detection of changes in function in early AD

Assuming that functional changes in AD are driven by cognitive decline, functional assessments are crucial to evaluating the impact of cognitive decline on the ability to carry out everyday activities. Both EMA and FDA guidelines emphasize the importance of outcome measures that assess meaningful cognitive function (with combined or separate assessment of cognitive and functional measures appropriate for use as primary endpoints in early AD), but neither guideline has provided the definition of “meaningful” (20, 43).

Functional abilities in IADL decline in early stages of AD due to the high level of cognitive ability associated with these activities, whereas BADL are affected later in the disease continuum (Figure 1B) (8); therefore, assessment of IADL is critical to detect any possible change in function in early stages of AD. Generally, tools have tended to focus on lower-level functioning or BADL. One exception is the ADCS-ADL-MCI, modified from the ADCS-ADL in order to emphasize IADL (Table 2A) (39). Likewise, the Amsterdam Instrumental Activities of Daily Living Questionnaire (A-IADL-Q) is sensitive to longitudinal change of IADL in patients with subjective memory complaints, MCI due to AD, and AD dementia (Table 2A) (35). Both tools align with the EMA recommendation to preferentially assess IADL over BADL (43). However, functional assessments are generally informant-based (8), which may not be the most appropriate approach in early stages of AD; in MCI due to AD, some degree of patient insight is generally preserved. Nonetheless, informant-based functional measures may be useful for longitudinal studies that extend beyond the point where patients can respond independently.

Executive function—a repertoire of cognitive processes used to control, regulate, and manage thoughts and behavior—is highly correlated with functional performance (51). A significant body of research has established that executive function assessment tools serve as a reliable proxy measure of functional skills (52, 53). For example, significant correlations between the NTB, which measures executive function and memory, and the Disability Assessment for Dementia (DAD), which measures functional abilities, (r=0.55) were observed in studies of lecozotan. Cognitive assessments that do not include robust measures of executive function, such as the widely used ADAS-Cog 11, do not tend to correlate highly with functional abilities (32).

#### Detection of changes in behavior in early AD

Currently, there are limited tools available for assessment of behavior across the AD continuum. One tool, used widely as a clinical trial endpoint, is the Neuropsychiatric Inventory Questionnaire (NPI-Q) (3, 54). This is a self-administered, informant-based questionnaire, developed to assess NPS in clinical practice. Derived from the original NPI-10 (54) the NPI-Q is widely used in both trials and practice, and assesses 12 neuropsychiatric domains, as well as informant distress levels (Table 2A). Although not designed specifically for early stages of AD, it has been used to detect NPS in patients early in the AD continuum (55). Patients who exhibited accelerated decline on the NPI-Q were more likely to develop dementia; thus, this tool may be useful in detecting changes in behavior earlier on in the course of disease.

Another informant-based assessment tool measuring NPS, extensively used in both research and clinical centers, is the Behavioral Pathology in Alzheimer’s Disease Rating Scale (BEHAVE-AD) (Table 2A) (54). Like all such measures, the BEHAVE-AD can be affected by informant status, such as recall issues or interpretation bias. The modified Empirical-BEHAVE-AD as well as the NPI-Clinician version (NPI-C) mitigate some of these problems by including direct observation of the patient’s behavior (54). However, in early stages of AD, patients themselves may be more insightful and knowledgeable than their informants regarding their own neuropsychiatric symptoms, including anxiety and apathy.

#### Detection of changes in early AD by composite or global scales

The FDA recognized the value of composite assessments for the evaluation of patients with MCI due to AD (54). Composite scales that include both cognition and function within a single scale may more effectively assess longitudinal changes than single measures, by enabling enhanced measurement of individual variability in clinical decline (34). In addition, composite assessments that include a semi-structured approach allow clinicians the flexibility to evaluate patients more comprehensively. Composite scores have increased power to detect change and may enable shorter or smaller trials. Although FDA guidance no longer recommends specific endpoints, it continues to emphasize the relevance of a link between cognition and function (20).

One measure explicitly identified in the FDA’s guidance was the CDR, which has served as a primary or secondary endpoint in multiple clinical trials (56). The CDR is a semi-structured interview that comprises 75 items relevant to cognition and function, takes ~25 minutes to administer, and is conducted sequentially with the informant and the patient (36). The CDR global score yields an overall rating of disease severity, while the CDR-SB score yields more detailed information on performance across six categories (three functional domains and three cognitive domains) (37). However, the use of CDR-SB in clinical practice may be limited by the subscales (three out of six) involving aspects of daily function (e.g., personal care) not usually impaired in early stages of the disease (57), the lack of behavioral items, and administration requirements (36).

A more practical alternative to the CDR-SB is the Cognitive-Functional Composite (CFC) that is comprised of seven cognitive tests of memory and executive function, plus the functional component represented by a shortened version of the A-IADL-Q (35). The CFC takes only ~25 minutes to administer. The CFC was recently shown to be sensitive to clinical progression in AD, specifically in early stages (34). Other composite scales that have shown sensitivity to changes in early stages of AD are the Alzheimer’s Disease Composite Score (ADCOMS), which incorporates items from the MMSE, CDR, and ADAS-Cog-12 (42), and the integrated Alzheimer’s Disease Rating Scale (iADRS), which combines scores from ADAS-Cog-14 and ADCS-IADL (58). For ADCOMS, individual measures are administered normally and select items are summed and weighted to generate the ADCOM score; the iADRS score is generated from a sum of the two component tools (42, 58).

### Additional considerations for detection of change in clinical features in early stages of AD

Shifts in cognition and function are subtle and decline is generally slow in the early stages of AD (25). To effectively assess change, assessment tools must be sensitive to subtle change and be conducted repeatedly and over a sufficient period of time (25, 59). This enables differentiation among periods of decline, stability, or improvement, to assess a therapeutic effect. While test frequency will depend on trial duration, as well as disease stage and measurement tool sensitivity, a challenge to conducting frequent assessments is the increased vulnerability to practice effects, which may result in improved scores over time masking treatment benefit. A key consideration for selecting endpoints such as the NTB and the CFC is that they are not prone to exhibit practice effects (60, 61).

Both patient and care partner must be considered when selecting clinical endpoints for early AD (62). The role of the care partner shifts across the AD continuum; in the early stages when the patient remains relatively independent, the care partner provides emotional support, facilitates tasks if needed, and assists in planning for the future in anticipation of disease progression; with greater patient decline the care partner provides more hands-on assistance (63). The What Matters Most study, part of the Alzheimer’s Disease Patient and Caregiver Engagement (AD PACE) initiative, found that while traditional cognitive assessments address a range of meaningful outcomes, they do not address outcomes like decreased socialization and mood-related symptoms highly valued by patients and care partners (27). Many of these changes, among others deemed important by patients and informants (64), are reflected in current measures (Table 2A, 2B) (65); nevertheless, a range of tools are required to capture the full spectrum of changes that are considered important by patients and informants.

As AD has marked impact on patients and their families, assessing QOL may enable an improved holistic understanding of the impact of AD and of potential therapeutic benefit. Indeed, the EMA recommends including secondary endpoints such as health-related QOL scales in clinical trials (43). In addition to the insight they provide researchers, these assessment tools provide key information to population-health decision makers, such as payers, policymakers, and advocacy organizations, regarding clinical meaningfulness of changes in disease severity (66, 67). In early AD, particularly during MCI due to AD, there is less impact on QOL and burden compared with AD dementia, due to a lesser impairment on cognition and function (11). Therefore, these assessment tools may be less sensitive to change in the disease’s early stages, compared with more advanced disease. QOL assessments must be interpreted carefully, as they measure many influences on QOL, including financial wellbeing, the strength of personal relationships, and the extent of a patient’s supportive social network, all pre-disease aspects of an individual’s life that are not directly affected by drug treatment assessed in trials. Nevertheless, using QOL measures and evaluating care partner burden in clinical trials, such as with the QOL in AD Scale (QOL-AD) or Zarit Burden Interview, may provide important insight into both patient and care partner (Table 2A).

Beyond measures of QOL, the pharmacoeconomic impact of therapy is often important to decisions of patient access. Additionally, functional and global outcomes—which are linked to costs, resource utilization, and care-partner burden—are valued by payers in assessment of a treatment’s cost effectiveness (67). Cost effectiveness is linked to overall health-system burden, which similarly influences the decisions of payers, regulators, and policymakers. The perspective of payers and other health decision makers, in addition to patients and their care partners, should be considered carefully when selecting endpoints for clinical trials.

## Are clinical trial endpoints applicable to clinical practice?

It is important that clinical trial endpoints capture data that are meaningful to clinicians, patients, and others AD stakeholders. Measuring the appropriate outcomes can instill clinicians with the confidence that an approved therapeutic for AD is beneficial for their individual patients. However, what works in clinical trials, both in terms of the tool and frequency of administration, may not necessarily work in practice (Table 2A, 2B) (68, 69). Ultimately, the choice of tool should be guided by researchers’ and clinicians’ differing needs and constraints. Nevertheless, some tools lend themselves more readily than others for use in both settings—the MMSE, MoCA, FAQ, and NPI-Q (Table 2A), for example—and leveraging such instruments in both contexts may provide a valuable continuum of insight from trial to clinic.

Despite the practical limitations such as cost, training requirements, and administration time, the tools used in clinical practice must measure cognition, function, and behavior to provide a comprehensive view into the disease and accurately assess and monitor patient status. Ideally, assessment tools used in clinical practice should be sensitive to the disease stage in which they are used. They should be brief and free of charge, should not require specialist personnel or extensive training, and, if possible, administered digitally (9). These characteristics are reflected in the various tools widely used in clinical practice today (Table 2A, 2B). A number of different approaches have been used to overcome some of the constraints with using structured instruments in clinical practice, such as shortening or translating tools, as seen with the NPI-Q (shortened from the NPI), which is suitable for both clinical trials and clinical practice (70). However, a battery specific to the restrictions of clinical practice, and mindful of patient, care partner, and system burden, would benefit the assessment of AD in this setting, as well as the evaluation of potential future therapeutics.

## Clinical meaningfulness

Clarifying the clinical meaningfulness of trial outcomes would promote understanding of the totality of benefits of treatment. Clinical meaningfulness is the practical importance of a treatment effect regarding its impact on the patient and/or family; this differs from statistical significance, which describes the probability of trial outcomes being due to random chance (71). Any clinically meaningful difference determined between groups must be statistically significant; however, alone, statistical significance between groups does not necessarily reflect clinically meaningful changes for patients (71–73). Clinical meaningfulness comprises two key elements: the relevance of the domains measured and the magnitude of treatment effect (74), and can be described either qualitatively or quantitatively. Qualitative clinical meaningfulness approaches, often assessed through semi-structured interviews or focus groups (75), involve gaining the opinion and experiences of patients, care partners, or clinicians (76). In contrast, quantitative clinical meaningfulness involves examination of thresholds of the minimal clinically important difference (MCID), also called minimal important difference (MID) (76, 77). Time to event analyses (described above) provide relevance and magnitude data to assess meaningfulness.

The MCID is the smallest change in an outcome measure score that leads to meaningful change for patients, which could be determined by patients, care partners, or clinicians. A difference between drug and placebo that is smaller than the MCID would not be considered clinically meaningful even if it were statistically significant, whereas a statistically significant difference that is at least as large as the MCID would be considered clinically significant. MCID values are highly sensitive and are dependent on the clinical trial population (and disease severity) to which they are derived and applied (76–78). MCIDs can also be used to examine individual-level score changes. For example, an individual patient whose score change is at least as large as the MCID can be categorized as a responder (65).

Several methods exist to calculate MCIDs – the two main methods involve a distribution-based approach or an anchor-based approach (74, 78). In the distribution-based approach, the MCID is determined entirely quantitatively, based on the effect size statistic, and is commonly defined as ½ standard deviation (or standard error of the mean) of the baseline outcome measure in the assessed population (73, 74, 78). The anchor-based approach involves anchoring a change in an outcome to a known meaningful change that may be based on previous research or opinion (73, 74, 77–79).

If the anchor-based approach is taken, the anchor must be carefully selected to ensure it is sensitive enough to detect differential levels of change, is relevant to the respondent’s perspective of clinically meaningful changes, and appropriate for disease stage. In earlier stages of AD, patients can report their symptomatic changes; in later stages, there is increased reliance on care partner’s and clinician’s report due to the patient’s lack of awareness that may compromise their ability to accurately report their symptoms (78). While several good anchors exist in AD, consensus is currently lacking (73, 77, 79). This is particularly challenging when considering whether to employ patient or care partner or clinical perspectives. A clear interpretation of the anchor value is needed to clarify the clinical meaningfulness scores (76). These challenges underscore the need to understand concepts of meaningfulness qualitatively and to apply these learnings when assessing clinically meaningful change. Examining concepts, behaviors, and activities that patients or care partners value, and considering impacts of intervention on the ability could add insights to an assessment (27, 64, 65).

### Defining clinically meaningful changes in early stages of AD

Defining clinically meaningful changes in early stages of AD is challenging due to the slow progression at this stage, as well as high levels of patient variability in symptom initiation and presentation (9, 23, 25). With disease-modifying therapies (DMTs), point difference and effect size may increase over time; thus, it is important to consider the duration of treatment necessary to achieve an effect. Given that clinical trials in early stages of AD have varying durations and use different scales, a consensus regarding treatment duration and point differences or effect sizes may be difficult to achieve (67). Furthermore, this should be considered along with changes in amyloid biomarkers or downstream biomarkers of intracellular tau pathology (phosphorylated tau) and neurodegeneration (total tau or neurofilament light) to establish the agent’s biological effect (8, 67). Considering such challenges, it is not surprising that currently there is no consensus on clinically meaningful changes in early stages of AD, with little quantitative research having been conducted on tools used across the AD continuum (74, 80–82).

#### Are existing definitions of clinically meaningful changes in AD applicable to early stages of disease?

Many MCIDs have been defined based on symptomatic changes, as with those reported in cholinesterase inhibitors (ChEI) trials (81, 83–85). One such study reported a 4-point change in ADAS-Cog as clinically meaningful at 6 months, on a group level, in patients with mild-to-moderate AD dementia (81). However, novel therapeutics may slow disease progression without evidence of symptomatic improvement (81). The previously published thresholds are mostly defined in late-stage patient populations, and as MCIDs vary for each instrument as well as according to disease stage or severity (80, 81, 83–86), this further limits their applicability to trial outcomes for therapeutics that potentially alter underlying disease pathology. One such therapy that has recently received accelerated approval by the US FDA is aducanumab, which selectively targets aggregated forms of amyloid beta (Aβ) comprising amyloid plaques (87, 88). Unlike other treatments for AD (i.e., ChEI and N-methyl-D-aspartate receptor antagonists), which are approved for mild or moderate-to-severe AD, the FDA-approved label specifies that treatment with aducanumab should be initiated in patients with the mild cognitive impairment or mild dementia stage of disease (87), for which more subtle effects are consistent with maintaining cognitive integrity, a key goal of early intervention (9, 23, 25). There is a need to modify existing thresholds that are based on later stages of AD and develop new measures to assess the clinical outcomes and meaningful effects of treatments approved for early stages of AD. This highlights the need for more stage-specific approaches for assessing and defining clinically meaningful changes in early AD.

The Insights to Model Alzheimer’s Progression in Real Life (iMAP) is one study aiming to address some of these gaps, by assessing the ability of the RBANS and Alzheimer’s Prevention Initiative Preclinical Composite Cognitive Test (APCC) to predict clinically meaningful outcomes, including a diagnosis of MCI due to AD or mild AD dementia, and changes in the CDR-SB (89). Research to examine MCIDs for differentially declining groups, based on natural history, have been reported for three assessment tools commonly used in clinical trials: the MMSE; CDR-SB; and FAQ (80). On average, meaningful changes were observed to be a 1–3-point decrease in MMSE, 1–2-point increase in CDR-SB, and 3–5-point increase in FAQ. The values reflect the range of meaningful change estimates across MCI due to AD; mild, moderate and severe AD dementia. These results should be interpreted with caution as The National Alzheimer’s Coordinating Center database used in the study may not be representative of the broader AD population, due to differences in patient characteristics and enrollment procedures (90). In addition, the diagnostic criteria for MCI due to AD did not reflect that of current clinical practice and a substantial population was not biomarker-verified. Further, study methods included several limitations. Primarily, the study used a binary anchor to assess meaningful changes in patients, and the extent to which this reflects a “minimum” is unknown. This study also relied only on clinician opinion, without patient or care partner viewpoint inclusion, potentially leading to a narrow view of disease progression.

Assessing trial outcomes via cognitive, functional, and behavioral changes, plus disease progression biomarkers, will allow better interpretation of an AD treatment’s clinical benefit (78). An example of a composite endpoint is the Preclinical Alzheimer Cognitive Composite, which detected subtle cognitive decline and an increased risk for progression to MCI or decline in CDR in clinically normal older adults with elevated Aβ biomarkers over three years (91). However, longer and larger trials may be required to detect clinically meaningful changes in cognition related to amyloid status, especially if cognition is the sole domain measured (92). Composite or global assessment tools such as the CDR may capture adequate cognitive and functional data to provide evidence of clinical meaningfulness. The FDA’s dual outcome criteria for trials is based on the core clinical features of cognition, plus a global or functional measure, to provide meaningful information (20). Therefore, using tools that are sensitive to early stages of AD for both cognition and function in clinical trials could facilitate conclusions regarding clinical meaningfulness in AD.

#### Additional challenges of defining clinically meaningful changes in early stages of AD

Additional challenges exist in quantitatively defining clinically meaningful changes in longitudinal data (76). Clinical meaningfulness definitions involve heavy reliance on patient, care partner, or clinician perception, and can involve retrospective recall, introducing of the potential for recall bias (76). Choosing a common mean from a wide distribution of change across the clinical trial population may mask patient variability, leading to possible reduced applicability of the final score to numerous patients (76). It can be informative to use the distribution-based approach to examine the proportion of patients who experience meaningful change, defined as at least ½ standard deviation (or standard error of the mean) of the outcome measure at baseline (73).

Variability of patients’ symptoms, in addition to differences in patients’ priorities in early stages of AD (Figure 2), complicate current attempts to use an MCID. For example, it may be meaningful for some patients to retain the ability to perform a task with reasonable success or efficiency, whereas for others, it is the ability to simply complete a task, even if they need to complete it differently from previously. Certain aspects of clinical features, including decline in memory and planning, and changes in behavior, were deemed highly important to patients and care partners in a recent AD PACE study (27). Therefore, defining clinical meaningfulness in these aspects is key to address the viewpoint of patients and care partners, and for investigation of the efficacy of future therapeutics.

### The need for consensus

The FDA states that clinical meaningfulness can be detected through changes in cognition, and it emphasizes the link between cognition and function (20). Both the EMA and FDA recommend that endpoints show clinically meaningful changes and note that novel assessment tools of AD are needed to provide evidence of this; neither agency provides a precise definition (20, 43). The FDA acknowledges that in early stages of AD (Stage 2 of their continuum [Figure 2]), it may be difficult to establish a clinically meaningful effect on subtle cognitive deficits within a clinical trial’s timeframe (20); additional longitudinal, real-world data collection, using assessment tools appropriate for clinical practice, may be beneficial.

Currently, there are differences in the interpretation of clinical meaningfulness from clinical trial data and real-world practice. Clear definitions, more comprehensive clinical meaningfulness discussions, and correlations between clinically meaningful differences for outcomes in AD clinical trials and clinical practice would provide key information regarding how a therapeutic may impact the everyday lives of the patients, their families, and care partners. Subsequently, this would also increase a clinician’s confidence in prescribing a therapeutic, add value to the payer for reimbursement, and help policymakers make informed decisions on the application of clinical trial outcomes in healthcare systems.

## Future perspectives

A number of assessment tools are currently in development or being validated that may provide increased sensitivity in the early stages of AD than current options (34). Several recommendations exist for an ideal endpoint for early stages of AD (Table 3), with a focus on longitudinal assessment of change with well-established psychometric and clinical validity (25, 68). Analysis approaches such as evaluating the drug-placebo difference over time (“area under the curve”) or time-to-event analysis may facilitate a quantitative assessment of the meaningful preservation of cognition and function.

Digital biomarkers hold promise as potential future endpoints for trial and clinical practice use, enabling passive, objective, large-scale data collection, with very low patient and researcher burden (59, 93, 94). They may capture data on potentially clinically meaningful changes in a patient’s day-to-day life (93), which are currently not captured in traditional measures (25, 94). These potential future biomarkers are subject to patient acceptance and ethical concerns; comprehensively evaluating and validating these is essential prior to clinical trial or clinical practice use (59, 94). Further use of current or future clinical trial endpoints, or potential digital biomarkers, could aid progress toward consensus on full, simple, and clear definitions of clinically meaningful changes. This would advance the field, given the potential emergence of AD therapeutics that alter underlying pathology.

## Conclusion

With the emergence of novel therapeutics for AD, it is increasingly important to ensure that assessment tools measure stage-specific, clinically meaningful changes in relation to AD progression and treatment response, focusing particularly on early AD. Progress in these efforts will improve patients’ expectations and knowledge of a prescribed therapeutic’s potential benefits to their everyday lives, and provide clearer understanding of an approved therapeutic’s socioeconomic relevance. Use of an appropriate battery that captures all aspects of the disease in clinical practice, while ensuring low burden on patients, care partners, and the health care system, will also aid in the assessment of the potential therapeutic benefit. Regulatory guidance is needed on outcome selection for AD clinical trials, especially those assessing therapeutics that potentially alter underlying disease pathology. This guidance should consider all stakeholders’ viewpoints to better generate meaningful data from trials. Support from real-world data and post-approval observations of effects of a therapeutic may provide key information on the applicability of trial results to a wider population.

## Figures and Tables

**Table 1.** Clinical features of AD: cognition, function, and behavior

Clinical feature	Domain	Description	Clinical presentation of domain in Alzheimer’s disease
Core cognitive memory domains	Episodic memory	Recollection of past events and personal experiences (Harvey 2019)	• Forgetting recent personal events (Alzheimer’s Association 2020) • Losing items around the home (Alzheimer’s Association 2020) • Forgetting recent conversations (Alzheimer’s Association 2020)
	Semantic memory	Knowledge of the world (facts, ideas, meanings, and constructs) and long-term storage of ideas and concepts (Harvey 2019)	• Difficulty naming an object (Alzheimer’s Association 2020) • Difficulty recognizing an object (agnosia) (Alzheimer’s Association 2020)
	Working memory*	A short-term storage module in which concepts are operated upon to solve problems (Harvey 2019)	• Impaired problem-solving skills (Alzheimer’s Association 2020) • Losing “train of thought” (Alzheimer’s Association 2020)
Other core cognitive domains	Executive functions	A set of processes used to control, regulate, and manage thoughts and behavior (Harvey 2019)	• Struggling to follow a series of steps (Alzheimer’s Association 2020) • Difficulty performing a familiar task (Alzheimer’s Association 2020) • Impaired judgment (Alzheimer’s Association 2020)
	Language	Verbal expression, language interpretation, and communication (Harvey 2019)	• Difficulty finding words (anomic aphasia) (Alzheimer’s Association 2020) • Struggling to follow a conversation (Alzheimer’s Association 2020)
	Visuospatial functions	The integration of space and visual form (Harvey 2019)	• Impaired perception of objects and faces (Alzheimer’s Association 2020) • Inability to navigate familiar surroundings (Alzheimer’s Association 2020)
Other cognitive domains	Attention*	The capacity to focus and sustain concentration (Harvey 2019)	• Impaired concentration (Alzheimer’s Association 2020) • Losing track of thoughts (Alzheimer’s Association 2020)
	Praxis	Monitor and process data in the surrounding environment to plan a motor action (Ward et al. 2015)	• Inability to perform a movement with a body part (Ward et al. 2015) • Inability to imitate a hand gesture (Ward et al. 2015)
Function	Basic activities of daily living	Ability to perform basic activities that are strongly correlated with motor functioning and coordination (Mlinac and Feng 2016)	• Reduced ability to carry out self-maintenance such as bathing, dressing, and grooming (Alzheimer’s Association 2020) • Reduced ability to carry out activities independently, such as walking and eating (Alzheimer’s Association 2020)
	Instrumental activities of daily living	Ability to perform complex activities, requiring greater cognitive organization than basic activities of daily living (Mlinac and Feng 2016)	• Reduced ability to manage finances or medications (Alzheimer’s Association 2020) • Reduced ability to shop, cook, conduct housework, or drive (Alzheimer’s Association 2020)
Behavior		Social behavior and psychological status (Kales et al. 2015; Alzheimer’s Association 2020)	• Neuropsychiatric symptoms such as aggression, anxiety, apathy, sleep disturbance, and psychosis (Kales et al. 2015; Alzheimer’s Association 2020)

* Working memory and attention are executive functions

**Table 2A.** A selection of assessment tools commonly used only in clinical trials or in both clinical trials and clinical practice

Assessment type	Assessment tool	Sensitivity and specificity for detecting AD dementia (unless stated otherwise)	Key advantages	Key limitations	Use across AD continuum				Setting	
					Stage 1/2	Stage 3	Stage 4/5	Stage 6	Clinical trials (1°/2°)	Clinical practice
					Pre-clinical AD	MCI due to AD	Mild-to-moderate AD dementia	Severe AD dementia		
Cognitive	ADAS-Cog-13/14 (Black et al. 2009; Sheehan 2012)	Not available	• Covers all cognitive areas in dementia	• Ceiling effects • Length of assessment makes unsuitable for clinical practice					Y - 1°	N
	CANTAB (De Roeck et al. 2019)	Sensitivity: 68% Specificity: 98%	• Automated test delivery and data capture • Language independent	• Does not include language-based measures • Fee for use					Y - 1°/2°	N
	CogState (De Roeck et al. 2019)	Sensitivity: 100% Specificity: 85%	• Automated test delivery and data capture	–					Y - 1°	N
	MMSE (Sheehan 2012; Cordell et al. 2013; Patnode et al. 2020)	Sensitivity: 89% Specificity: 89%	• Minimal training requirements	• Floor and ceiling effects • Fee for use					Y - 2°	Y
	MoCA (Sheehan 2012; Patnode et al. 2020)	Sensitivity: 78–100% Specificity: 65–94%	• Minimal training requirements • Sensitive to early stages of AD	• Floor effects at moderate AD					Y - 2°	Y
	NTB (Black et al. 2009; Schneider and Goldberg 2020)	Not available	• High test-retest reliability • Uses pre-baseline exposure to tests to induce stability	–					Y - 1°	N
	RBANS (Duff et al. 2008; Black et al. 2009)	Sensitivity: 98% Specificity: 82%	• Minimal practice effects • Translated and validated in multiple languages	–					Y - 1°/2°	Y
Functional	ADCS-ADL (Black et al. 2009)	Not available	• Graded responses allow gradual transitions to be detected	• Reliant on reliable informant					Y - 1°/2°	Y
	ADCS-ADL-MCI (Pedrosa et al. 2010; Marshall et al. 2012)	Sensitivity for MCI: 87% Specificity for MCI: 87%	• Suitable for MCI	• Reliant on reliable informant					Y - 1°/2°	Y
	A-IADL-Q (Sikkes et al. 2013; Koster et al. 2015)	Sensitivity: 74% Specificity: 65%	• Sensitive to changes in IADL functioning over time	• Reliant on reliable informant					Y - 1°/2°	Y
	FAQ (Marshall et al. 2012; Patnode et al. 2020)	Sensitivity: 90% Specificity: 83%	• High reliability	• Reliant on reliable informant					Y - 2°	Y
Behavioral	BEHAVE-AD/Empirical-BEHAVE-AD (Sheehan 2012; Ismail et al. 2013)	Sensitivity: 79% Specificity: 73%	• Empirical-BEHAVE-AD created to avoid bias	• Length of assessment makes less suitable for clinical practice					Y	N
	NPI-Q (Sheehan 2012; Ismail et al. 2013)	Sensitivity: 86% Specificity: 76%	• Short • Reliable across different languages and cultures	• Reliant on reliable informant					Y - 2°	Y
Composite/Global	ADCOMS (Wang et al. 2016; Schneider and Goldberg 2020)	Sensitivity: 82%	• May enable small sample sizes to show drug-placebo difference	• No alternate forms • Psychometrics not yet known					Y - 1°	N
	iADRS (Schneider and Goldberg 2020)	Not available	• Sensitive to tracking disease progression	• Minimal use as prospective clinical endpoint					Y - 1°	N
	CFC (Jutten et al. 2020)	Not available	• Moderate-to-high test-retest reliability • Minimal practice effects	• No current evidence for utility in subjective cognitive decline					Y	N
	CDR-SB (Black et al. 2009; O’Bryant et al. 2010; Marshall et al. 2012; Sheehan 2012)	Sensitivity: 74% Specificity: 81%	• Short • Good inter-rater reliability	• Length of assessment makes less suitable for clinical practice • Ceiling effects in early stages of AD					Y - 1°/2°	N
	CIBIC Plus (Sheehan 2012)	Not available	• Comprehensive	• Training required • Reliant on reliable informant					Y - 1°	N
Patient-reported outcome	GAS (Black et al. 2009)	Not available	• Individualized for patient; • Can detect longitudinal changes; • High importance to patients and caregivers	Not widely favored for use in clinical practice	–	–	–	–	Y - 2°	N
Patient and caregiver QOL/burden	EQ-5D (Sheehan 2012)	–	• Short • Completed by patient or informant	• Reliant on reliable informant	–	–	–	–	Y - 2°	Y
	QOL-AD (Sheehan 2012)	–	• Extensively validated • Disease specific • Completed by patient and caregiver	• Informant version reliant on reliable informant	–	–	–	–	Y - 2°	Y
	Zarit Burden Interview (Sheehan 2012)	–	• Disease specific	• Length of assessment is restrictive	–	–	–	–	Y - 2°	Y

Abbreviations: 1° = primary, 2° = secondary, AD = Alzheimer’s disease, ADAS-Cog = Alzheimer’s Disease Assessment Scale–Cognitive Subscale, ADCOMS = Alzheimer’s Disease Composite Score, ADCS-ADL = Alzheimer’s Disease Cooperative Study–Activities of Daily Living Scale, ADCS-ADL-MCI = Alzheimer’s Disease Cooperative Study–Activities of Daily Living Scale–Mild Cognitive Impairment, A-IADL-Q = Amsterdam Instrumental Activities of Daily Living Questionnaire, BEHAVE-AD = Behavioral Pathology in Alzheimer’s Disease, Rating Scale, CANTAB = Cambridge Neuropsychological Test Automated Battery, CDR-SB = Clinical Dementia Rating–Sum of Boxes, CFC = Cognitive-Functional Composite, CIBIC Plus = Clinician’s Interview Based Impression of Change with caregiver input, EQ-5D = EuroQol 5-Dimension Questionnaire, FAQ = Functional Activities Questionnaire, GAS = Goal Attainment Setting, IADL = instrumental activities of daily living, iADRS = integrated Alzheimer’s Disease Rating Scale, MCI = mild cognitive impairment, MMSE = Mini-Mental State Examination, MoCA = Montreal Cognitive Assessment, NPI-Q = Neuropsychiatric Inventory Questionnaire, NTB = Neuropsychological Test Battery, QOL = quality of life, QOL-AD = Quality of Life in Alzheimer’s Disease Scale, RBANS = Repeatable Battery for the Assessment of Neuropsychological Status.

**Table 2B.** A selection of assessment tools commonly used only in clinical practice

Assessment type	Assessment tool	Sensitivity and specificity for detecting AD dementia (unless stated otherwise)	Key advantages	Key limitations	Use across AD continuum			
					Stage 1/2	Stage 3	Stage 4/5	Stage 6
					Pre-clinical AD	MCI due to AD	Mild-to-moderate AD dementia	Severe AD dementia
Cognitive	Clock-drawing test (Sheehan 2012)	Sensitivity: 86% Specificity: 96%	• Fast, simple • No training requirements • Minimal language requirement	• Assesses narrow part of cognitive dysfunction				
	GPCOG (Cordell et al. 2013; Patnode et al. 2020)	Sensitivity: 82% Specificity: 83%	• Minimal training requirements • Brief	• Informant version reliant on reliable informant				
	Mini-Cog (Cordell et al. 2013; Patnode et al. 2020)	Sensitivity: 88% Specificity: 71%	• Easy to interpret • No training requirements • Brief	• Relatively low sensitivity and specificity				
	MIS (Cordell et al. 2013; Patnode et al. 2020)	Sensitivity: 73% Specificity: 93%	• Free to use • Minimal training requirements	• Lacks information on executive functions or visuospatial functions				
Functional	DAD-6 (de Rotrou et al. 2012)	Sensitivity for MCI: 83% Sensitivity for MCI: 84%	• High discriminative ability to distinguish MCI from mild dementia	• Reliant on reliable informan				
Behavioral	GDS (Sheehan 2012)	Not available	• Reliable	• Validated in mild dementia but not moderate-to-severe dementia				

Abbreviations: AD = Alzheimer’s disease, DAD-6 = Disability Assessment for Dementia Scale, GDS = Global Depression Scale, GPCOG = General Practitioner Assessment of Cognition, MCI = mild cognitive impairment, MIS = Memory Impairment Scale.

**Table 3.** Recommended requirements of an ideal assessment tool for use in early stages of AD in clinical trials and/or clinical practice

Ideal properties of an assessment tool	
1	Be sensitive to subtle changes in early stages of AD
2	Be able to track impairment longitudinally and be sensitive to changes over time
3	Be able to show clinically meaningful changes regarding progression of AD
4	Have well-established psychometric and clinical validity
5	Have minimal or no range restrictions such as floor and ceiling effects
6	Have minimal practice effects
7	Be coherent with biomarker changes
8	Be cross-culturally applicable or have alternate forms, each with well-established psychometric and clinical validity
9	Be able to provide valuable information to the patient, family, caregiver, primary care provider, and payer

Abbreviation: AD = Alzheimer’s disease.

## Abbreviations:

1°: primary
2°: secondary
Aβ: amyloid beta
AD: Alzheimer’s disease
ADAS-Cog: Alzheimer’s Disease Assessment Scale–Cognitive Subscale
AD PACE: Alzheimer’s Disease Patient and Caregiver Engagement
ADCOMS: Alzheimer’s Disease Composite Score
ADCS-ADL: Alzheimer’s Disease Cooperative Study–Activities of Daily Living Scale
ADCS-ADL-MCI: Alzheimer’s Disease Cooperative Study–Activities of Daily Living Scale–Mild Cognitive Impairment
ADL: activities of daily living
A-IADL-Q: Amsterdam Instrumental Activities of Daily Living Questionnaire
APCC: Alzheimer’s Prevention Initiative Preclinical Composite Cognitive Test
ApoE: apolipoprotein E
BADL: basic activities of daily living
BEHAVE-AD: Behavioral Pathology in Alzheimer’s Disease; Rating Scale
CANTAB: Cambridge Neuropsychological Test Automated Battery
CDR-SB: Clinical Dementia Rating–Sum of Boxes
CFC: Cognitive-Functional Composite
ChEI: cholinesterase inhibitor
CIBIC Plus: Clinician’s Interview Based Impression of Change with caregiver input
DAD-6: Disability Assessment for Dementia
DMT: disease-modifying therapy
EMA: European Medicines Agency
EQ-5D: EuroQol 5-Dimension Questionnaire
FAQ: Functional Activities Questionnaire
FDA: Food and Drug Administration
GAS: Goal Attainment Setting
GDS: Geriatric Depression Scale
GPCOG: General Practitioner Assessment of Cognition
IADL: instrumental activities of daily living
iADRS: integrated Alzheimer’s Disease Rating Scale
iMAP: Insights to Model Alzheimer’s Progression in Real Life
MCI: mild cognitive impairment
MCID: minimal clinically important difference
MID: minimal important difference
MIS: Memory Impairment Screen
MMSE: Mini-Mental State Examination
MoCA: Montreal Cognitive Assessment
NPI-C: Neuropsychiatric Inventory Questionnaire–Clinician version
NPI-Q: Neuropsychiatric Inventory Questionnaire
NTB: Neuropsychological Test Battery
QOL: quality of life
QOL-AD: Quality of Life in Alzheimer’s Disease Scale
RBANS: Repeatable Battery for the Assessment of Neuropsychological Status
